# DNA polymorphism underlying allozyme variation at a malic enzyme locus (mMEP‐2*) in Atlantic salmon (*Salmo salar* L.)

**DOI:** 10.1111/jfb.15182

**Published:** 2022-08-14

**Authors:** John B. Taggart, Michael J. Leaver, Michaël Bekaert

**Affiliations:** ^1^ Institute of Aquaculture, Faculty of Natural Sciences University of Stirling Stirling Scotland UK

**Keywords:** allozyme, malic enzyme, population, selection, SNP

## Abstract

A non‐synonymous single nucleotide polymorphism (SNP) underlies a diallelic allozyme polymorphism at the mitochondrial NADP‐dependent mMEP‐2* locus in Atlantic salmon (*Salmo salar* L.). The resultant amino acid substitution, which alters the charge of the allelic products, matches the differential mobility of the two allozyme alleles, whereas allozyme and SNP assays revealed genotyping concordance in 257 of 258 individuals. A single mismatch, homozygous allozyme *vs.* heterozygote SNP, suggests the presence of a second, less common null allele.

The study of loci under selection can shed light on adaptive evolutionary processes and may be particularly informative for population delineation and management (Powers & Schulte, 1998; Watt, 1994). The nuclear DNA encoded mitochondrial NAPD‐dependent malic enzyme locus mMEP‐2* in Atlantic salmon, *Salmo salar* L., exhibits a diallelic allozyme polymorphism (alleles *100 and *125), which is variable throughout the species' geographic range (Bourke *et al*., 1997). A number of population‐based studies have highlighted mMEP‐2* allele and genotype frequencies that are indicative of a selection signal (*e.g*. Gilbey *et al*., 1999; Jordan *et al*., 1990, 1997; Moran *et al*., 1998; Verspoor & Jordan, 1989). Further research has stalled, however, with population genetic studies now using direct DNA marker screening platforms. To prime further research into mMEP‐2* the authors report on the identification of a causal SNP (accession ss9410532730) for the observed mMEP‐2* allozyme polymorphism and the development of simple rapid DNA‐based assays to survey it.

Frozen skeletal muscle tissues for both allozyme and DNA screening were opportunistically acquired from archived materials, collected over a 19‐year period (1997–2015) from a range of unrelated projects. These comprised 258 individuals (including pedigree parents detailed later) from six different sources: two rivers (Dee and Tay, Scotland *n* = 36 and 32, respectively), three commercial European farm strains (*n* = 50, 40 and 40) and a ranch strain (Burrischoole, Ireland *n* = 60). In addition, archived DNA samples from wild populations inhabiting the latitudinal limits of the species range were also surveyed: two rivers from Finnmark, Norway (*n* = 46 each; Kongsfjordelva and Repparfjordelva) and two rivers from northern Spain (*n* = 30 each; Rio Ulla and Rio Bidasoa). Sampling was conducted according to national regulations in place at the time the specimens were taken.

The gene search focused on archived DNAs from two *S. salar* mapping panels (Br5 and Br6) originally generated from an EU‐funded linkage mapping project (SALMAP, 1997–2000). The salmon families interrogated were outcrosses involving four wild‐caught adults (River Tay, Scotland), each pedigree comprising sire, dam and 48 progeny. Three allozyme loci, including mMEP‐2*, were found among the *c*. 350 markers, mostly short tandem repeats (STRs), assigned to a low‐resolution map based on these pedigrees (Danzmann *et al*., 2005).

Starch gel electrophoresis of skeletal muscle extracts resolves three phenotypes, indicative of polymorphism at mMEP‐2* involving two alleles (*100 and *125; Figure 1a) as previously described (Cross *et al*., 1979). Variability at mMEP‐2* proved to be informative for both pedigrees (Br5 – sire *100/*125 × dam *100/*100; Br6 – male *100/*125 × *100/*125). Screening of the progeny confirmed Mendelian inheritance in both pedigrees.

**FIGURE 1:**
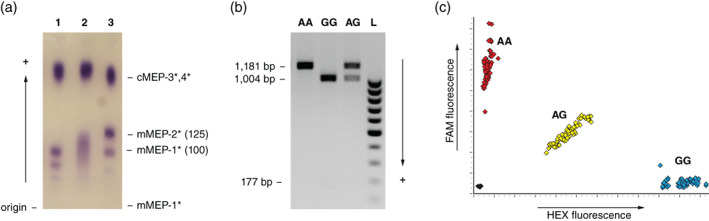
Screening of MEP‐2* polymorphism. (a) Starch gel zymogram of muscle extract, 1 = *100/*100; 2 = *100/*125; 3 = *125/*125; (b) SNP RFLP analysis, *Bsa*HI digests of exon 8–10 amplicons (small 177 bp band usually very weak); (c) KASP assay, plot of fluorescently tagged alleles

The preliminary search for MEP genes was conducted in 2012. Using malic enzyme vertebrate homologue sequences (mouse and zebrafish) as a starting point, NCBI and EBI databases (plus salmonid TIGR and GRASP EST repositories – no longer accessible) were interrogated to identify potentially relevant mRNA sequences in *S. salar*. For clarity, the gene locations and mRNA sequences for *S. salar* reported here are referenced to the latest (May 2022) Atlantic salmon genome assembly (Ssal_v3.1, NCBI; RefSeq GCF_905237065.1), and genes are referred to by allozyme nomenclature. Multiple BLASTn/x analyses identified two main mitochondrial‐type MEP mRNAs (XM_014164545.2 and XM_014174329.2) and their genes identified (LOC106581960, on chromosome 21 and LOC106586750 on chromosome 25, respectively). These were assumed to represent the expected duplicate pair of mMEP* enzyme loci. Using a draught genome, kindly provided by Ben Koop, STR loci located within intronic regions of both genes were identified and screened in the pedigrees (see Supporting Information Table S1 for details). Independent segregation (*P* = 0.8) was found between the LOC106581960 STR and mMEP‐2*. In contrast, there was complete co‐segregation between LOC106586750 STR alleles and mMEP‐2* alleles (*i.e*., zero recombinants among the 96 progeny), identifying LOC106586750 as the mMEP‐2* gene, whereas LOC106581960 is likely to be mMEP‐1*.

The mMEP‐2* gene (30,884 bp) comprises 15 identified exons (3853 bp), with the amino acid coding sequence (CDS) comprising 614 codons – spanning mid‐exon 2 to mid‐exon 15. A set of eight PCR assays were devised to allow sequencing of the entire CDS region (Supporting Information Table S2). Full CDS sequencing of two progeny from family Br6 (alternate allozyme homozygotes *100/*100 and *125/*125) was undertaken. Only a single point mutation difference in the CDS was found between the two individuals, a non‐synonymous A→G substitution (*100 and *125 alleles, respectively) in exon 10 (position 1273 of XM_014174329.2; Supporting Information Figure S1). This produces a charge‐changing amino acid replacement at codon 371 – asparagine (N, no charge; *100 allele) for aspartic acid (D, negative charge; *125 allele), consistent with faster electrophoretic movement of the *125 allele products towards the positive pole. The mutation sits within the NADP+ binding domain of the enzyme. This polymorphism has been captured in accessions, the amino acid substitution being the only difference between the original protein reference sequences XP_014029804.1 (D) and its recently updated replacement XP_014029804.2 (N).

The identified SNP (NC_059466.1:40628412:A:G, SNP accession ss9410532730) occurs within the restriction site of restriction enzyme *Bsa*H1, allowing RFLP‐based detection, *e.g*., using the exon 8–10 primer set (Table 1). The SNP “A” base disrupts the restriction enzyme site (no cutting), whereas the “G” base permits restriction, yielding 1004 and 177 bp products (Figure 1b). A fluorescent allele‐specific PCR assay (KASP; LGC Genomics) was also designed as a rapid screening tool (Figure 1c; Table 2).

**TABLE 1:** RFLP assay primer details. Amplicon size: 1181 bp, PCR primer annealing temperature: 62°C

Name	Primer Sequence (5′ → 3′)
Ex8‐10.F	TTTGACTATCTGACCGACCGTTCAC
Ex8‐10.R	CTCGCCTTAATAGGTGTGCGTTTCT

**TABLE 2:** KASP allele‐specific primer details

Name	Primer sequence (5′ → 3′)^a^	Fluorescent tail
F_MEP2.A	gaaggtgaccaagttcatgctGGAGGGAGTGTCCGCCACAA	FAM
F_MEP2.G	gaaggtcggagtcaacggattGAGGGAGTGTCCGCCACAG	HEX
R_MEP2	GATTAGCTATGATATTATGGTATTGCAGTT	

*Note*. Amplicon size: 146 bp (including tails; PCR primer annealing temperature: 65°C→57°C (Touchdown, KASP standard protocol).

^a^ Bases in uppercase letters are locus specific, whereas lowercase tails allow for incorporation of the complementary fluorescent tags present in the KASP mastermix.

In total 258 individuals from six different populations/stocks were screened for both allozyme and SNP variability (identifying 61, 124 and 73 as *100/*100, *100/*125 and *125/*125 allozyme “genotypes,” respectively). Polymorphism was observed in all six stocks. There was almost complete concordance between allozyme and SNP scoring (257 of 258 individuals). A single fish was scored as *100/*100 for allozyme and AG with both RFLP and KASP assay. The entire CDS of this individual was sequenced, confirming the AG genotype. No other sequence differences were found. A null allele, caused by mutation in a regulatory gene region, could account for this observation.

Four population samples from both latitudinal range extremes were screened for SNP variability. Contrasting frequencies for the “A” allele (*100) were observed between northern samples (*f*_(*A*)_ = 0.48 and 0.33; Kongsfjordelva and Repparfjordelva, respectively) and southern samples (*f*_(*A*)_ = 1.00 and 0.67; Ulla and Bidasoa, respectively).

Converting targeted allozyme polymorphisms to DNA‐based assays has many practical advantages. Integration with high throughput modern screening platforms is made possible, sample storage is simplified and ethical concerns relating to large‐scale surveys can be addressed through non‐invasive sampling. Furthermore, temporal studies can be expanded by both screening of curated samples and the assimilation of existing allozyme datasets. The approach has been used for salmonid studies in the past (*e.g*., Brunelli *et al*., 2008; McMeel *et al*., 2001).

With genomic resources for species expanding at a rapid rate, the identification of DNA mutations underlying allozyme variation is becoming more straightforward. In the current study, the use of mapping pedigrees to confidently distinguish between duplicate loci was particularly helpful. The sole point mutation within the CDS of alternate allozyme homozygote sibs, causing a charge changing amino acid substitution matching electrophoretic expectations, is compelling evidence that this is a causal SNP. Furthermore, there was extremely high concurrence between allozyme and SNP assays from the same individuals (>99.5%), and similar allele frequency disparity was observed for both SNP assay (this study) and allozyme assay (Verspoor *et al*., 2005) among populations from extremes of the *S. salar* latitudinal range. Both observations lend support for the SNP polymorphism being a robust proxy for previously reported mMEP‐2* allozyme polymorphism.

A single mismatch, homozygous allozyme *vs*. heterozygote SNP genotype, was observed. This could be indicative of an additional low frequency “null” allele, which could potentially generate a false selection signal in samples, if present at a higher frequency. Nonetheless, a null allele, which should lead to an overestimation of homozygote numbers, would not clearly explain the excess of allozyme heterozygotes found among grilse reported by Jordan *et al*. (1990). Further work is needed to clarify this issue. There is a large array of potential regulatory mechanisms that can underlie null alleles (Rojano *et al*., 2019), which can be extremely difficult to identify solely from DNA sequence data (*e.g*., Saha *et al*., 2022).

This characterised DNA polymorphism and assay will allow for more intensive work into the widely suspected selective action at mMEP‐2*. As well as the single locus assays described above, it should be straightforward to include this assay into existing bespoke SNP panels used routinely for population and pedigree screening, and it is already represented multiple times on an existing Atlantic salmon high‐density genotyping array (Houston *et al*., 2014).

## AUTHOR CONTRIBUTIONS

J.B.T.: conception, data generation, data analysis and manuscript preparation. M.J.L. and M.B: data analysis and manuscript preparation.

## FUNDING INFORMATION

This research did not receive any specific grant from funding agencies in the public, commercial or not‐for‐profit sectors. The fees for article processing charges were covered by the University of Stirling APC fund.

## CONFLICT OF INTEREST

The authors declare no conflict of interest.

## ETHICAL STATEMENT

No samples were specifically collected for this project, and archived material was repurposed. Sampling was conducted according to national regulations in place at the time the specimens were taken. Abidance to animal welfare laws, guidelines and policies as approved by University of Stirling Ethics Committee (Animal Welfare and Ethics Review Board; AWERB 2022 5001 6348).

## Supporting information


**APPENDIX S1** Supporting Information
