# Supplementary material for: DNA polymorphism underlying allozyme variation at a malic enzyme locus (mMEP‐2*) in Atlantic salmon (Salmo salar L.)
Source: J Fish Biol. 2022 Aug 14;101(5):1371–4. doi: 10.1111/jfb.15182 (PMC9804884; doi:10.1111/jfb.15182)
Supplement: Supplementary file 1 — APPENDIX S1 Supporting Information [file JFB-101-1371-s001.pdf]

DNA polymorphism underlying allozyme variation at a malic enzyme locus (mMEP-2*) in Atlantic salmon (*Salmo salar* L.)

SUPPLEMENTARY MATERIAL

Supplementary Figure S1

mMEP-2* exon 10 partial sequences detailing the non-synonymous SNP mutation.

Supplementary Table S1

Details of PCR primers for microsatellite loci located within targetted draft genome contigs and subsequent locus identification. PCR primer annealing temperature: 63°C.

Supplementary Table S2

Exon coding positions and PCR primer details for CDS sequencing.

SUPPLEMENTARY FIGURE

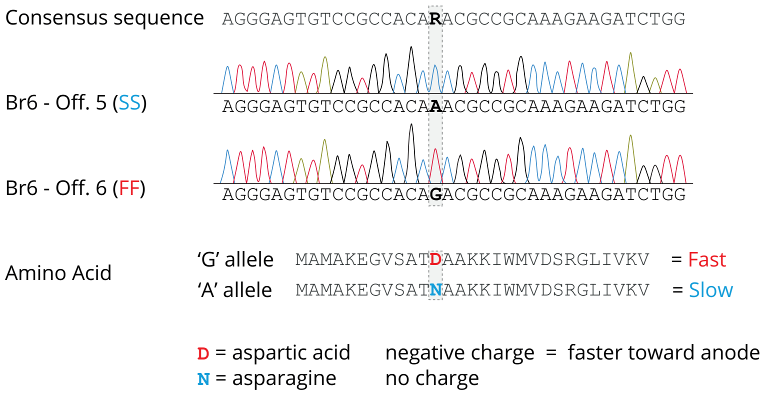

Supplementary Figure S1. mMEP-2* exon 10 partial sequences detailing the non-synonymous SNP mutation.

SUPPLEMENTARY TABLES

Supplementary Table S1. Details of PCR primers for microsatellite loci located within targetted draft genome contigs and subsequent locus identification. PCR primer annealing temperature: 63°C.

Name	Primer Sequence (5′ → 3′)	STR motif	Allozyme co-segregation	Isozyme locus	Gene ID
P1_For	ACCCCCTGTACATCGGTCTAAAG	$(GT)_{25}$	NO (P = 0.8)	mMEP-1*	106581960
P1_Rev	TGCAGCGCAGAGTAGTGAACG				
P2_For	TCGGCCTGAAGCATAAGAGGAT	$(GT)_{27}$	YES (P = 0.0)	mMEP-2*	106586750
P2_Rev	AAGGCGTTGGAGTTGGCAAAGT				

Supplementary Table S2. Exon coding positions and PCR primer details for CDS sequencing.

	Exon Location						Intronic PCR details for full CDS sequencing					
	mRNA		Gene								Location in gene	
Exon	Start	End	Start	End	Codon size	Amplicon	Primers	Primer Sequence (5′ → 3′)	Amplicon size	T_a	Start	End
1	1	190	1	190	-	-						
2	191	411	793	1014	216 bp		Ex2.F	TGTGGAGCTCCTGCACCTAA	487 bp	60°C	671	1157
							Ex2.R	TGGAGCTGATCTGCTGGTGT				
3	412	542	18819	18950	129 bp		Ex3.F	AGGCCTAATAAAGGAATGCTAAAGA	472 bp	60°C	18621	19092
							Ex3.R	TCGTGCAGTGATTAGTGATAATGAG				
4	543	692	20158	20307	150 bp		Ex4.F	CCAGCTCTGTTTCACCTTGTCCTAC	656 bp	62°C	19872	20527
							Ex4.R	CTTCAACTGTGCATGCTAACACTTC				
5	693	768	21854	21929	75 bp							
6	769	930	22079	22240	162 bp		Ex5-7.F	GCGAGTGCCTTTTGAATTTTGATAC	1,059 bp	62°C	21791	22849
7	931	1034	22616	22719	105 bp		Ex5-7.R	TGCAGCGCAGAGTAGTGAACG				
8	1035	1144	22851	22960	108 bp							
9	1145	1242	23440	23537	99 bp		Ex8-10.F	TTTGACTATCTGACCGACCGTTCAC	1,181 bp	62°C	22811	23991
10	1243	1356	23750	23863	114 bp		Ex8-10.R	CTCGCCTTAATAGGTGTGCGTTTCT				
11	1357	1462	26053	26158	105 bp		Ex11.F	CCTTGTGAACTGGATCCAGTAGTAT	377 bp	60°C	25991	26367
							E11.R	ATGGGGATCCTAATAAAATACCATC				
12	1463	1605	27766	27908	144 bp							
13	1606	1779	28128	28301	174 bp		Ex12-14.F	GGCAAATCTGATGGCCGAATGGTA	1,088 bp	62°C	27701	28788
14	1780	1878	28613	28711	99 bp		Ex12-14.R	AAATCGGCGTGGAGGTGGGAACT				
15	1879	3886	28942	30951	162 bp		Ex15.F	TCCCACCTCCACGCCGATTTG	416 bp	62°C	28769	29184
							Ex15.R	TTTCTGATATGTGGCTCCCTTCTGC				
